# Supplementary material for: Evaluation of oat genotypes for species-specific and cross-resistance to Fusarium species and the role of trichomes in susceptibility
Source: Front Plant Sci. 2025 Aug 12;16:1608030. doi: 10.3389/fpls.2025.1608030 (PMC12378175; doi:10.3389/fpls.2025.1608030)
Supplement: Supplementary file 1 [file DataSheet1.docx]

Supplementary Material

## Supplementary Figures


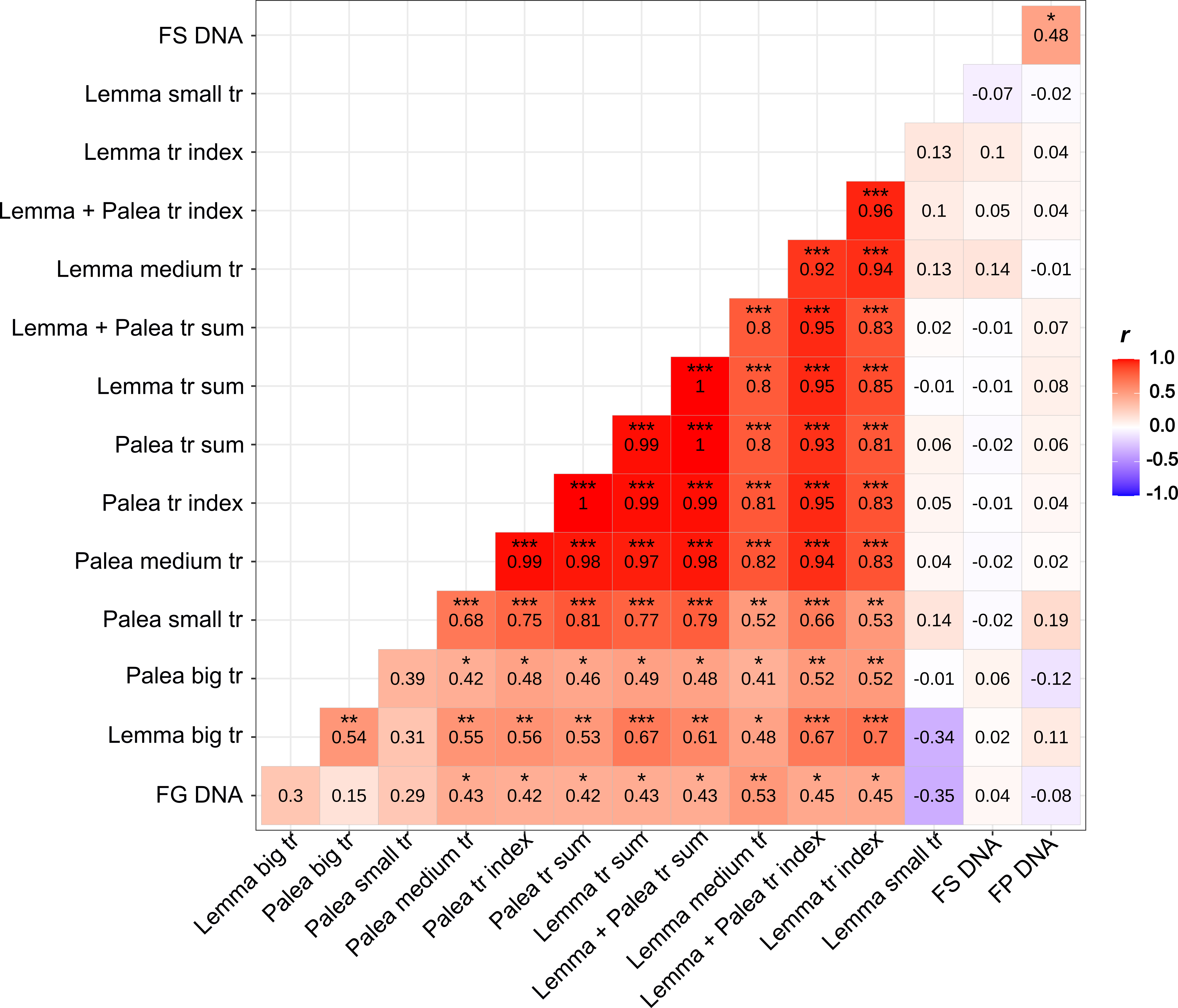


Supplementary Figure 1. Correlation matrix of trichome counts (sum) and trichome indices and *Fusarium* biomass. Fungal biomass was assessed via quantification of fungal DNA per kg oat seeds. BLUEs were used for calculation of Pearson correlation coefficients. Asteriscs indicate p value significance levels with p < 0.001: ***; p < 0.01: **; p < 0.05: *. tr: trichome; FG: *F. graminearum*; FP: *F. poae*; FS: *F. sporotrichioides; r*: Pearson correlation coefficient.


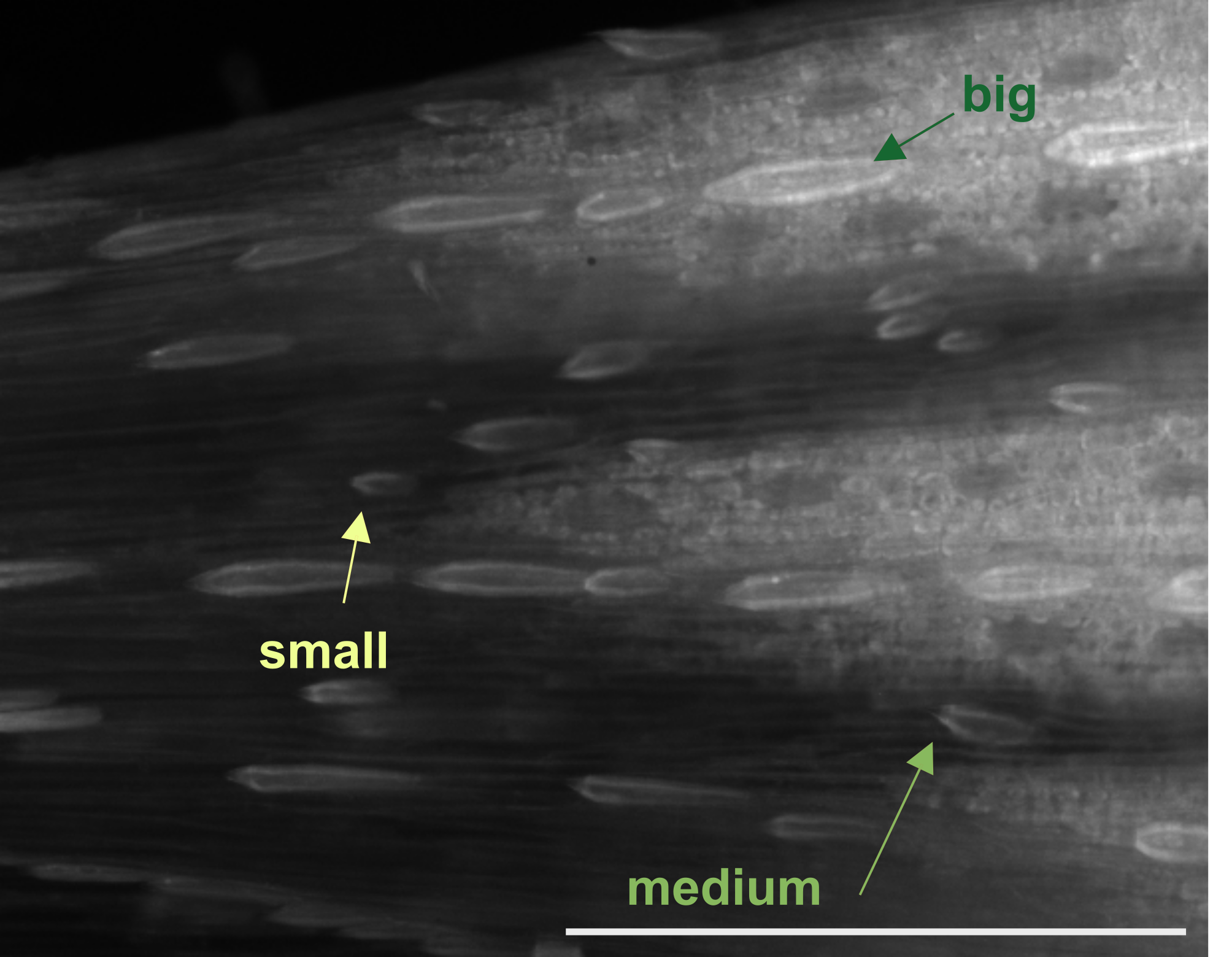


Supplementary Figure 2. Examples of trichomes for each size class. Scale bar: 500 µm.


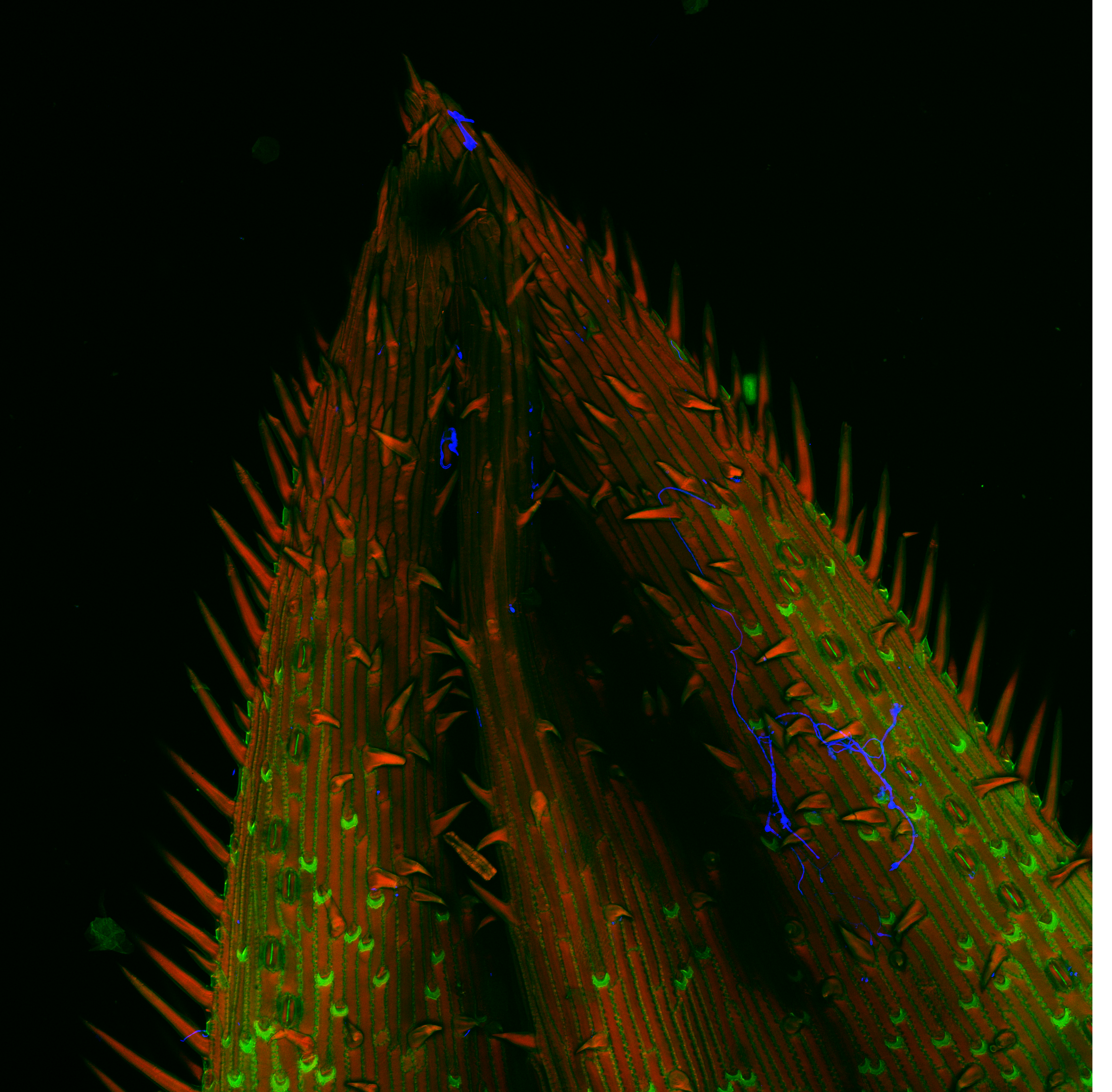


Supplementary Figure 3. Overview image of *F. graminearum* growth on oat palea 96 hpi. Fungal hyphae were stained using WGA-AF488 and are shown in blue. Maximum projection of CLSM overlay images. hpi: hours post inoculation.


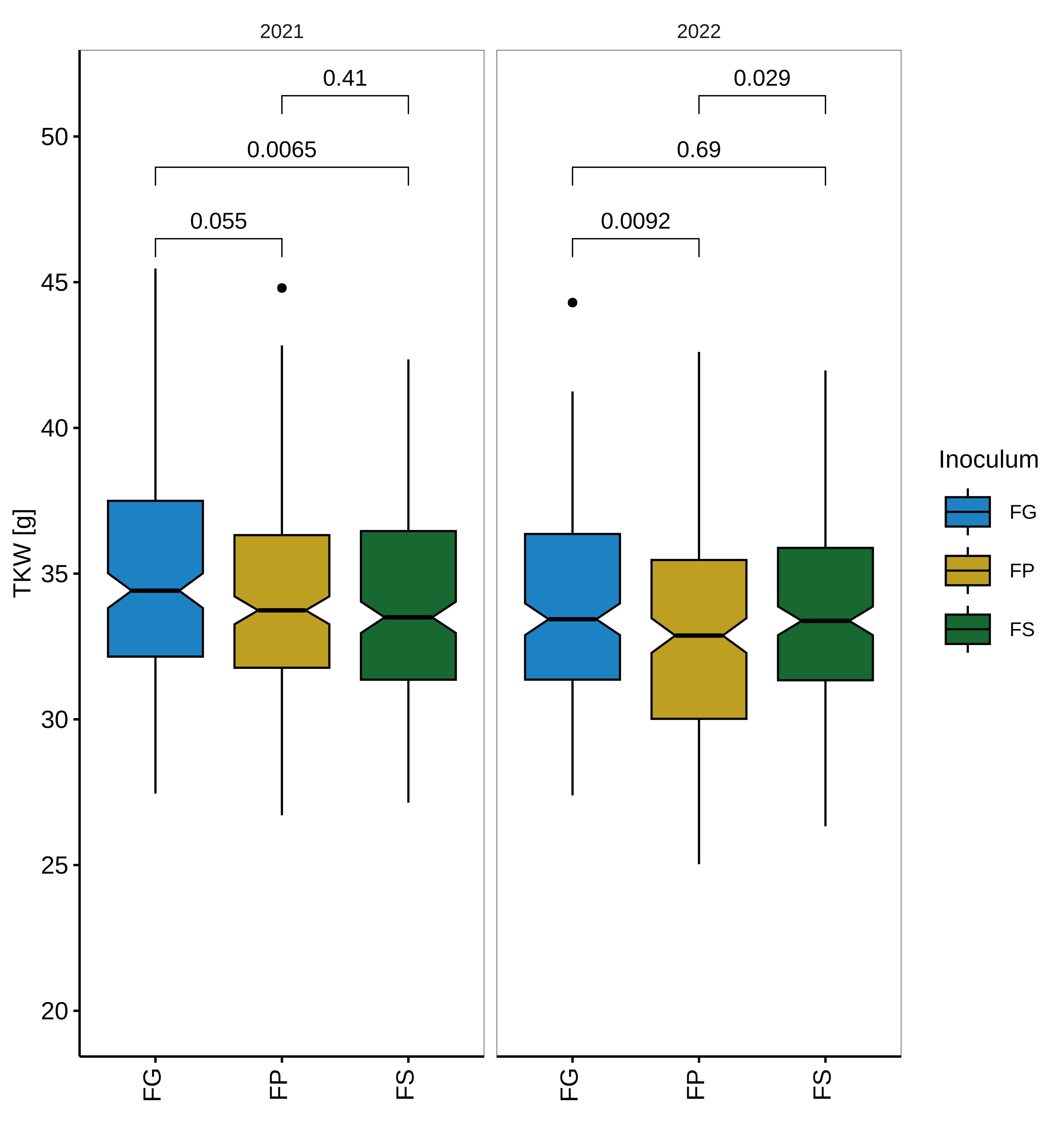


Supplementary Figure 4: Thousand kernel weight (TKW) in [g] across oat genotypes for the different inoculi and years. Wilcox-p-values are given at the top. FG: *F. graminearum*; FP: *F. poae*; FS: *F. sporotrichioides.*
